# Climate Change and Health Preparedness in Africa: Analysing Trends in Six African Countries

**DOI:** 10.3390/ijerph18094672

**Published:** 2021-04-27

**Authors:** Samuel Kwasi Opoku, Walter Leal Filho, Fudjumdjum Hubert, Oluwabunmi Adejumo

**Affiliations:** 1 Research and Transfer Centre “Sustainable Development and Climate Change Management”, Hamburg University of Applied Sciences, Ulmenliet 20, D-21033 Hamburg, Germany; opoku8600@gmail.com (S.K.O.); fudjumh@yahoo.fr (F.H.); 2 Institute for Entrepreneurship and Development Studies, Obafemi Awolowo University, Ile-Ife 220282, Osun State, Nigeria; adejumobum@oauife.edu.ng

**Keywords:** climate change impacts, health professionals, health systems, preparedness African countries

## Abstract

Climate change is a global problem, which affects the various geographical regions at different levels. It is also associated with a wide range of human health problems, which pose a burden to health systems, especially in regions such as Africa. Indeed, across the African continent public health systems are under severe pressure, partly due to their fragile socioeconomic conditions. This paper reports on a cross-sectional study in six African countries (Ghana, Nigeria, South Africa, Namibia, Ethiopia, and Kenya) aimed at assessing their vulnerabilities to climate change, focusing on its impacts on human health. The study evaluated the levels of information, knowledge, and perceptions of public health professionals. It also examined the health systems’ preparedness to cope with these health hazards, the available resources, and those needed to build resilience to the country’s vulnerable population, as perceived by health professionals. The results revealed that 63.1% of the total respondents reported that climate change had been extensively experienced in the past years, while 32% claimed that the sampled countries had experienced them to some extent. Nigerian respondents recorded the highest levels (67.7%), followed by Kenya with 66.6%. South Africa had the lowest level of impact as perceived by the respondents (50.0%) when compared with the other sampled countries. All respondents from Ghana and Namibia reported that health problems caused by climate change are common in the two countries. As perceived by the health professionals, the inadequate resources reiterate the need for infrastructural resources, medical equipment, emergency response resources, and technical support. The study’s recommendations include the need to improve current policies at all levels (i.e., national, regional, and local) on climate change and public health and to strengthen health professionals’ skills. Improving the basic knowledge of health institutions to better respond to a changing climate is also recommended. The study provides valuable insights which may be helpful to other nations in Sub-Saharan Africa.

## 1. Introduction

There is clear evidence that climate change is affecting countries around the world and that it poses a threat to the achievement of the UN Sustainable Development Goals (SDGs) [1]. Current systems in place aimed at implementing the SDGs (especially SDGs 6, 7, 13, 14, and 15) have prioritised tackling issues that address climate change and environmental sustainability [1]. The fourth and fifth assessment reports issued by the Intergovernmental Panel on Climate Change (IPCC) emphasised the need for stakeholders (i.e., governments, policymakers, and academics) to take urgent actions on climate change to ensure sustainable development. Recent studies have shared a similar agreement [2,3]. The United Nations Framework Convention on Climate Change through the Paris Agreement [4] has emphasised the need to reduce CO2 emissions as one of the measures to tackle the problem. 

From a regional perspective, some of the fundamental drivers of climate in Africa are the Inter-Tropical Convergence Zone (ITCZ), the El Niño—Southern Oscillation (ENSO) and the West African Monsoon (W.A.M.) [5,6]. Climate distortions in the W.A.M. lead to changed wind and precipitation conditions in Africa’s tropical and sub-tropical regions. This provides favourable conditions for the transmission of vector-borne diseases [7]. The World Health Organization [8] identified Africa as the region most affected by malaria. Although significant measures to reduce the incidence of malaria in Africa are in place, the Institute of Health Metrics and Evaluation (IHME) and WHO estimated that nine in every ten deaths from malaria are from the African region [9].

Another index of environmental distortions is the warming of the earth’s climate, which creates offshoots of ITCZ and ENSO movements. The evidence of these movements appears principally in Southern Africa and the Sahelian part of Western Africa, where there are increases in sea and air warming, which result in heavy rainfall and floods [10,11]. These, in turn, are associated with losses of agricultural produce, which can cause famine, increases in the mortality rate, and increased exposure to infectious diseases, often with an increased exposure to toxic substances [12,13]. 

The United Nations (UN) reported in 2017 the impact of severe flooding in the West and Central Africa axes [1]. The incident resulted in acute human and physical losses, reflected in river overflows, displacement of people, and an increase in disease burdens and deaths in Sierra Leone, Guinea, Mali, Ghana, Nigeria, Burkina Faso, the Central African Republic, and Niger. In Niger, the official estimate of flood victims was 206,513, with 56 deaths, 1200 houses damaged, 16,000 herds of cattle lost, and 9800 hectares of cultivated land lost. The case of Nigeria was not so different, with more than 100,000 people affected by about 21 local government areas. Similarly, almost all the Burkina Faso regions were affected, with 30,862 people impacted by flooding and violent winds [1].

In the Eastern part of Africa, where dry weather is prevalent, there have also been drought experiences due to the ITCZ-ENSO movements [6]. ENSO’s progress reflects the processes that occur in the Pacific [5]. However, its impacts—which are in the form of increases in surface air temperature, and sea and land warming—gives rise to drought and water unavailability, which have spill over effects in Africa and particularly in Eastern Africa. Historically, the El Niño between 1997and 1998 resulted in droughts and forest fires in some Asian economies and in East Africa, where many health challenges were also recorded [6]. According to a study [13], the drought areal-extent decadal spans from 0.6% in Tanzania to 3.7% in Ethiopia. In contrast, the probabilities of experiencing drought can be as high as 40%, and most of the dryness in East Africa for 11 decades (1903–2013) spans between 14 and 24 months over two years.

Indeed, Africa is one of the most vulnerable regions to climate variability and change [14]. The literature identified some of the effects of the climate alterations in Africa, such as sea-level rise, glacier melting, water resources reduction, reduction in agricultural production and food security, lesser biodiversity increases in zoonotic diseases, and increase in erosion drought, and flood [6,15,16]. An increase in sea and land temperatures is an expression of the growth of warming, despite identifying water bodies, such as seas and oceans, as absorbers for climate distortions or ensuring eco-balance [17].

An increase in greenhouse gas discharges reflects increases in human socioeconomic activities [18]. These socioeconomic activities mainly depend on utilising environmental resources, which distort certain climatic conditions, such as the earth’s energy balance, which creates severe health implications. This distortion has resulted in two global goals: reducing greenhouse emissions and improving air quality. Evidence has shown that the air quality in many urban areas in Africa is the leading cause of respiratory disorders, such as lung cancer, asthma, dementia, chronic obstructive pulmonary disease, and cardiovascular diseases [7].

An increase in vector-borne diseases, such as malaria, lymphatic filariasis, onchocerciasis, schistosomiasis, African trypanosomiasis, and Rift Valley fever, is linked to climate change impacts. These diseases are, therefore, higher in developing economies than in developed economies [19]. Apart from the low-income challenge, poor health, and environmental conditions in Africa, vector-borne diseases directly bear on tropical climate areas, typical of the sub-Saharan African climate [19].

Climate change worsens the already precarious situation seen in low-income countries’ public health systems [20]. Africans are undoubtedly witnessing these pressures and even surprises from climate disorder dimensions, especially human health and sustainability [12]. Public health planners, policymakers, and other relevant stakeholders must understand how climate change will affect public health in their area to enable them to address such issues.

Globally, the impacts of climate change on public health are inconsistent. This inconsistency is because of geographical variations and the varying capacities of countries to adapt to these changes. Sensitisation on the part of the representatives of public health systems on the health impacts of climate change is needed [20]. Therefore, to effectively address these impacts, the various health systems need to understand and consider the relationship between climate change and human health, sharing this knowledge with the relevant stakeholders. The experience and sharing of knowledge regarding how climate change will affect human health will increase the preparedness of policymakers to respond to increases in the prevalence of some diseases. Therefore, this study highlights aspects of climate change experienced, using the sampled African countries, and their impact on human health. The study also assesses the extent of vulnerability to climate change impacts on human health and the health systems’ perception of them. Furthermore, this study determines the health systems’ preparedness in the sampled African countries to cope with climate change’s health impacts on human health.

Other areas of interest within the study include assessing the governments’ financial (budget) responses seen in the sampled African countries to deal with the observed human health impacts of climate change, the effectiveness of the available resources, and those needed to cope with the effects of climate change on human health. This study also aims to contribute to better policies to improve present and future generations’ livelihoods, despite the variabilities in Africa’s climate conditions. The overall aim of the study was to assess the preparedness of health systems, based on the responses by the surveyed respondents, who work in these countries and know the extent to which they are prepared to cope with the pressures posed by climate change. The use of the opinion of the respondents cannot be regarded as the sole criteria for the assessment of preparedness; however, it is an important one to the extent that such witness statements are unbiased and reflect the reality, being more reliable than government statements, which sometimes do reveal real trends.

### Health Impacts of Climate Change in Africa

Conforming to the Intergovernmental Panel on Climate Change (IPCC) report, climate change impacts on Africans’ health have been quite enormous, especially for vulnerable groups, such as the poor, women, and children [19,21,22]. The challenge of water, food and vector-borne diseases, malnutrition, and mental health consequences can be attributed mainly to extreme weather events, temperature and rainfall patterns, thereby posing a health threat to the African continent [23].

Several studies have documented the impacts of climate change on health [19,21,24,25]. Direct effects include the following: extreme events-related injuries and mishaps; infectious diseases associated with water, vector biology and food contamination; manifestations of allergies coupled with increased allergen productions; air pollution causing respiratory and cardiovascular diseases; and malnutrition related to food insecurity [24,26]. Mental health consequences, population migration, and civil conflicts are among other indirect health effects for which data to assess their magnitude of occurrence are limited [24]. The U.C.L. Lancet Commission reports that climate change impacts account for 34% of the global disability-adjusted life years (DALYs) in Sub-Saharan Africa. The burden of the health risk associated with climate impacts is reported as three times as great in the worldwide population since Sub-Saharan Africa only accounts for 11% of its population [27].

Climate change will increase people’s exposure to water-related contaminants and cause illness by affecting fresh and marine water resources. Bacteria, viruses, protozoa, and other blue-green algae are pathogens and toxins that cause water-related diseases [28]. 

A review of cholera seasonality suggests a long association with climate change and poor sanitation. The outbreak of cholera is associated with heavy rainfall and floods, particularly in Ghana [29]; Nigeria [30]; and El Nino-Southern Oscillation in Southern [23] and Eastern [31] African countries. In Ghana, Namibia, South Africa and Ethiopia, precipitation and temperature increase the number of diarrhoeal cases, resulting in many deaths [32,33,34,35].

Other climate-sensitive water-borne diseases reported in many parts of the continent with severe health outcomes are the Buruli ulcer and schistosomiasis, occurring in Ghana [36] and Nigeria [37]. 

Malaria, leishmaniasis, Rift Valley fever, and tick and rodent-borne diseases are the most frequently occurring vector-borne diseases, causing myriads of premature morbidity and mortality rates in Africa [23]. As one significant disease associated with climate change [27], malaria varies regionally with temperature, with its highest occurrence at 25 °C and falls above 28 °C [23]. Studies investigated correlations of rainfall pattern, temperature variability, humidity, land and sea surface temperature and total precipitation with malaria in Africa [38,39,40,41]. The findings showed that temperature and precipitation patterns play a role in neglected tropical diseases, such as leishmaniasis and schistosomiasis. Leishmaniasis occurs mainly in Northern African countries with recent emergence in western regions due to co-infection. Meanwhile, changes in rainfall pattern are causing Rift Valley fever in the Horn of Africa. In addition, there is a rise in tick distribution and tick-borne diseases in Eastern and Southern Africa due to land use and cover change; prolonged Harmattan periods across Africa engender favourable meningitis conditions [23,30,42].

Further studies explored the health impacts of climate change on nutrition and food security across Africa. Stunted growth and underweight conditions among children in Mali, Kenya, Ethiopia, and Ghana (grey literature) due to malnutrition and food insecurity have a significant association with the severity of drought, floods, and extreme temperature [23,43,44,45,46]. Despite the availability of food sources and nutritional value in parts of Africa, low transportation networks due to floods hamper food distribution [47]. 

A study concluded that temperature and moisture influence fungal growth and aflatoxin production in cereals and legumes. Their presence inhibits infants’ growth due to the consumption of contaminated food ingredients [48]. The delayed and sharp decrease in rainfall gives way to drought and decreased crop yields, culminating in malnutrition in the savanna zones in countries like Ghana. The population livelihood depends on rain-fed agriculture [49].

The severity of atmospheric warming causes low crop production, which has, in turn, increased food insecurity and shifted the population’s attention to imported food, which affects the livelihood of the vulnerable in Nigeria, South Africa, and Ghana [50,51,52,53], as well as causing malnutrition among children in Ethiopia [54]. A study in Ethiopia concluded that about 2.7 million people required emergency food assistance in 2014, while 238,761 children had treatment for severe and acute malnutrition [54].

Some other studies extensively reviewed the health impacts of extreme weather events on the African continent. The effect of floods from severe precipitation caused numerous fatalities and victims’ enforcement to leave their homes in the Eastern African countries, Central and Western Africa [20,25,55,56,57,58]. Some impacts of mental health consequences from severe climate events are anxiety, depression, social dysfunction, and loss of confidence as part of the health effects that beset the surviving populace [59,60,61,62,63]. Heatwaves and heat-related health effects from extreme temperatures recently gained attention in Africa through increasing related death tolls. Heat rashes and multiple stressors in West African countries, involving Ghana, Nigeria, Burkina Faso, and Kenya in the East, and Zimbabwe and South Africa in the South [21,64,65,66,67,68], with children being the most affected [23]. Maintaining work levels and output due to extreme temperature decreases the agricultural workload in African countries. For instance, in a study conducted in South Africa, the reduced work capacities and outputs were attributed to heat waves, which caused severe sunburns, sleeplessness, irritability, and exhaustion in workers [69]. Risk assessments were conducted for extreme health impacts across African countries, especially Kenya, both at the regional and city scale. The findings showed that 10,000 to 86,000 people would be affected by severe floods by 2030, which will cost the country between ZAR 7 and ZAR 58 million [70]. 

**Figure 1:**
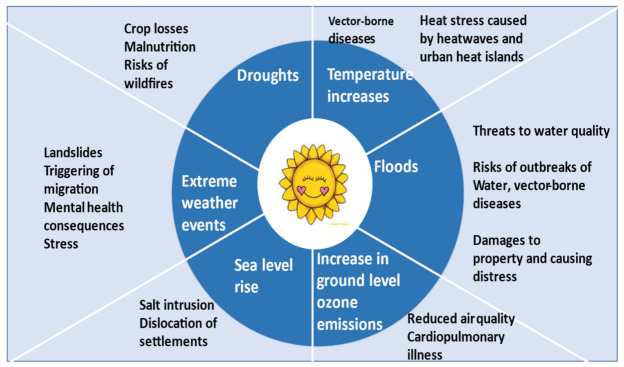
Relations between climate change and some of its health impacts. Source: authors.

From Figure 1 while some impacts are directly influenceable (e.g., malnutrition from droughts), others are indirect (e.g., mental health problems due to dislocation or losses in crops or livestock).

## 2. Methodology

**Figure 2:**
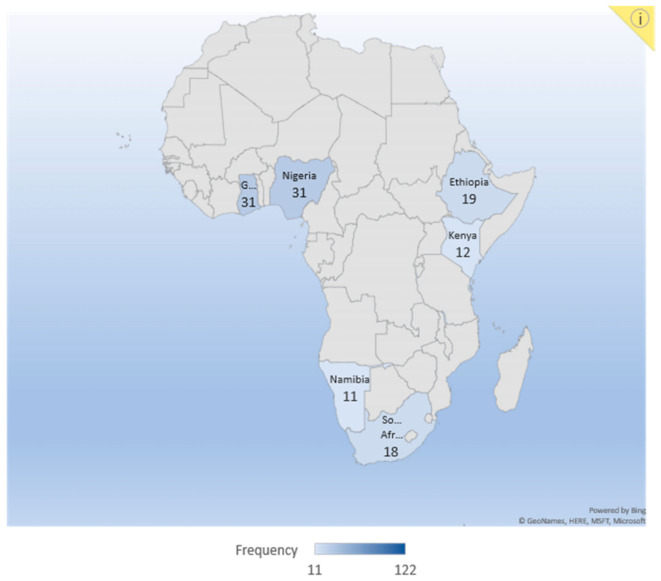
Africa map highlighting the countries studied.

A cross-sectional study was conducted in six (6) different African countries to understand the country’s vulnerability to climate change impacts on human health and assess their health systems’ preparedness towards these impacts. The selected countries were (1) Ghana and (2) Nigeria, in West Africa; (3) Namibia and (4) South Africa, in Southern Africa; and (5) Kenya and (6) Ethiopia in East Africa (see Figure 2). The population of each sampled country according to Worldometer in 2020 is 31,072,940 in Ghana; 206,139,589 in Nigeria; 59,308,690 in South Africa; 2,540,905 in Namibia; 114,963,588 in Ethiopia; and 53,771,296 in Kenya, respectively [71]. These 6 countries represent the various African regions because of their high vulnerability to climate impacts. 

### 2.1. Data and Sample

#### 2.1.1. Secondary Data

The authors searched for and tabulated some of the existing peer-reviewed literature (Table 1) from each sampled country between 2014 and 2019 through PubMed and Google Scholar. The search for evidence focused on five significant climate change impacts on human health from each sampled country: (1) impact on water-borne, water, and sanitation diseases; (2) impact on vector-borne diseases; (3) extreme weather events impact; (4) impacts on nutrition, food security and distribution; and (5) impacts on mental health and wellbeing. This was done to determine each country’s vulnerability to the impacts, their exposure to climate drivers, the study location within the country and the potential resulting outcomes from the exposure to enable authors to measure sampled country’s public health system’s preparedness towards these challenges through our survey.

#### 2.1.2. Primary Data Collection

The authors employed a questionnaire-based survey to complement the literature on the vulnerability of the sampled countries to climate change impacts. The survey again aims to assess these countries’ health systems’ readiness to cope with climate change impacts. Primary data were collected between April and the first week of June 2019 through an online survey using a structured questionnaire with several closed-ended and very few open-ended questions (see Appendix A). In the sampling stage, the authors purposely selected professionals in the field of health from government agencies, higher institutions, non-governmental agencies, and research institutions in each sampled country and reached out to them via e-mail contacts (see Table 2). The expected sample size from each participant country was about 30. Typically, it was widely disseminated in such surveys but attracted the interest of participants who had a substantial interest in the topic.

One advantage of the sampled professionals is that they have expertise in climate change impacts on human health and improve resilience through adaptive measures. The authors divided the questionnaire into five (6) main parts. The first part addressed the background information of the respondents with their corresponding country. The second part examined the respondents’ knowledge and perception of climate variability and human health impacts in each sampled country; part three of the research question assesses each country’s vulnerability to climate change effects. Section four assesses the health professionals’ perception of the health systems’ preparedness in dealing with the health impacts of climate change in each country. The fifth section assesses the available interventions in dealing with the issues of climate change effects. The sixth section evaluates each sampled country’s available resources to deal with climate change impacts on human health and the health professionals’ resources to curb the effects. The authors analysed the survey data using IBM SPSS statistics 25, and the findings are presented in the subsequent parts of this paper.

## 3. Results

All the research questions relating to the study are presented in this section. Their discussion is followed in the next section. Tables are predominantly used in the results to give precise percentages. The necessary details of the respondents (e.g., workplace with their position) are described. Information on climate change in the sampled countries, with its impacts on human health, as observed by respondents, is also presented. Other analyses tabulated include respondents’ perceptions of health professionals’ preparedness to cope with climate change’s health hazards. Finally, the health resources available and those needed to curb the challenges of climate change impacts on public health are also described.

### 3.1. Respondents Background Information

A purposive sampling technique that targeted only professionals in the health systems at various sectors (see Table 3) with prior knowledge on climate change and its impacts on human health were selected. In the selection process, these professionals were professors, coordinators, consultants, public health administrators, directors, project safety supervisors, lecturers, students, the health ministries from sampled countries, climate change institutions, nurses, senior researchers, etc. All respondents from the sampled countries obtained their weather/climate information from meteorological agencies, radio/television stations, internet sources, as well as from different data providers (G.C.M. model outputs).

### 3.2. Respondents Knowledge and Perception of Climate Change and Health Impacts

It can be seen in Table 4 that all respondents knew the potential health impacts of climate change of which examples were given as follows:

- Infectious diseases related to changes in vector biology, water, and food contamination.
- Injuries and fatalities related to extreme weather events and heatwaves.
- Allergic symptoms related to increased allergen production.
- Mental health consequences, civil conflicts, and population dislocation.

Our survey results showed that climate change had extensively been experienced in the past years, as reported by 63.1% of respondents. In comparison, 32.0% claimed that the sampled countries had experienced them to some extent. Nigeria was perceived to have the highest climate experience (67.7% within the country), and Kenya (66.6% within the country). South Africa had the lowest climate change experience as perceived by the respondents (50.0% within the country) compared to other sampled countries.

Respondents from West Africa (Ghana, Nigeria) reported that floods and extreme temperatures are frequent, and those from Eastern Africa (Ethiopia, Kenya) mentioned drought and extreme temperatures. Extreme temperatures appear to be common in the Western and Eastern part of Africa. 

The Southern African countries (South African and Namibia) experienced much drought and extreme temperatures. To some extent, floods and erratic rainfall pattern were observed in all the sampled countries.

These responses confirm a reviewed article, *Strengthening climate change adaptation capacity in Africa- case studies from six major African cities and policy implications* [77], which indicated that cities in African countries have been vulnerable to climate change impacts.

### 3.3. Public Health’ Vulnerability to Climate Change Impacts as Perceived by Respondents in Sampled Countries

About half (50.8%) of the respondents revealed that the health impacts of climate change had been extensively observed in the sampled African countries, while 38.5% of the respondents reported that the effects were experienced only to some extent. All respondents from Ghana and Namibia (100%) revealed that the health problems caused by climate change are common in the two countries. In contrast, only 27.8% of South African respondents showed that climate change was observed. Malaria and other vector-borne diseases were reported in both Ghana (67.7%) and Nigeria (64.5%) as most prevalent. Nigeria and Ethiopia reported considerable percentages of food-borne illnesses, malnutrition, and food insecurity (48.4% and 57.9%). 

The respondents’ perceptions regarding the trend of the diseases from the past until now were examined, as shown in Table 5. The table reveals that diseases caused by climate change have extensively been increased, with Nigeria having the highest percentage (80.8%) in the magnitude and severity of the disease. Though South Africa had the lowest percentage increase (66.7%), it is significant to conclude that sampled African countries face a significant challenge to infections caused by climate change impacts.

In total, 76.4% of total respondents predicted that the magnitude and severity of climate-induced diseases in sampled countries would increase in the following years if climate change were not accounted for in the planning and implementation of public health programs.

### 3.4. The Health Systems’ Preparedness in Dealing with the Health Impacts of Climate Change as Perceived by Respondents

The implications regarding the sampled country’s preparedness to effectively respond to climate change’s health impacts are presented in Table 6 to assess the necessary intervention to improve coping and adaptation strategies. The table indicates a severe deficit in all aspects of preparedness in the sampled countries to effectively respond to climate change impacts. Percentage differences in the impacts’ preparedness priority between the sampled countries, the insufficient healthcare professionals without enough training regarding climate change, and the lack of delivery systems to address health problems caused by climate change impacts are significant evidence of the ill-prepared situations of the sampled countries to improve on the coping and adaptation strategies effectively. 

### 3.5. Available Interventions in Sampled Countries as Perceived by Respondents in Dealing with Health Problems of Climate Change Impacts

Table 7 reveals some intervention programs to deal with climate change impacts as perceived by respondents. The table indicates that all sampled countries have programs to address water and vector-borne diseases and control, programs to secure the public health livelihood, especially the vulnerable ones in times of extreme events, and the coping and adaptation plan to build the country’s resilience regarding climate change impacts. Their effectiveness remains a big challenge, as seen in the table. All the respondents revealed considerable percentages to define the ineffectiveness of the existing intervention programs in each country. Some of the available programs mentioned by the respondents include the following: the ‘WASH’ campaign program to deal with water provision, sanitation and hygiene, borehole drilling, an emergency management agency, a climate-resilient water safety plan, environmental sanitation–drainage operations, a mosquito and vector spraying project, a planting for food and jobs program, climate watch and predictions, awareness creation using radio and television, disease and emergency notifications, afforestation programs to reduce the risk of extreme events, carbon dioxide removal and risk management, the National Disaster Management Organization (NADMO), flood warnings and a temporary shelter program in coastal and flood-prone areas, a South African expert on nutrition, food security/distribution during a famine, community awareness and a sensitisation program.

Respondents’ perceptions of disaster relief agencies in the sampled countries are presented in Table 8. Although these agencies/organisations exist in the sampled African countries, their practical implementation on a wide range of climate crises becomes challenging. It is seen from the table that those that exist are insufficient in operation in times of heavy impacts, such as flooding. Some of these existing organisations, as mentioned by the respondents, are as follows:

The National Disaster Management Organization (NADMO); The United Nations International Children’s Emergency Fund (UNICEF); The National Emergency Management Agency; USAID; World Vision; ADRA, National Disaster Prevention and Management Agency; Disaster Risk Reduction Commission; The Red Cross; Gift of the Givers, providing support in drought-prone areas; NEMA; LASEMA; The AYA Foundation; EHPs Disaster Risk Management; Meals on Wheel (N.G.O.); Public Health Emergency and Preparedness Agency; Catholic Relief Agency; and F.A.O.

The financial response from the sampled country’s government and the needed funding/budget at the health sectors as perceived by the respondents are presented in Table 9. A total of 83.9% of responses, especially from Ghana, revealed that climate change is not well incorporated into public health intervention. A total of 77. 4% perceived that the intervention programs available are technically not well supported by the Ghanaian government. Due to this, 90% perceived the need for funding in the health systems’ resources to sufficiently build resilience to climate change anomalies, particularly for vulnerable populations. Compared with South Africa, respondents revealed a lower percentage, partly due to their excellent health systems managed by the government. The percentage differences in Table 7 and Table 8, on account of South Africa performance in times of climate change crises, are too low and, as such, confirm the assertion that the health system preparedness in response to climate change impacts with their support from the government is, in one way or the other, equipped with the necessary resources compared to other sampled African countries. However, 77.8% of responses still claimed that the health sector needs additional resources to fully prepare themselves for future uncertainties posed by a changing climate, as shown in Table 10.

### 3.6. Resources Availability in the Sampled African Countries and Those Needed to Deal with Climate Change Impacts as Perceived by Respondents

Respondents reacted to the resources available within each sampled country to respond to climate change health problems in Table 10. Respondents further mentioned the country’s additional resources needed to ultimately reduce the change impacts and build resilience, particularly for the vulnerable populations. From the table, despite some of the countries’ performance (South Africa, Ghana, Namibia), all the sampled countries have limited resource availability (higher than 60%). This confirms respondents’ perceptions of the escalation of health problems resulting from climate change impacts because of the unavailable resources in the health systems to effectively deal with the increasing prevalence of diseases and other uncertainties. Most of the respondents (higher than 60%) from each sampled country suggested that for their country to equip themselves fully and thoroughly prepare for current and future health hazards posed by a changing climate, the health systems need to construct more hospitals and healthcare centres. These must include all hard-to-reach communities in the country where the most vulnerable populations are. When these hospitals and healthcare centres are adequately resourced with medical equipment, sufficient health professionals in all fields with enough training, particularly in the mental health field, are needed to take care of victims of climate change impacts.

Having prepared for curative measures, most of the respondents further perceived preventive steps to control future climate change impacts by having an effective surveillance system in the country. When a coordination framework exists between the health systems and the meteorological department to access relevant weather and climate data, the health system plans and implements health programs towards future uncertainties. Again, South African surveillance systems undoubtedly perform better than other countries (44.4%).

More so, almost all the responses mentioned that all the sampled African countries needed emergency response resources. The health systems’ preparedness towards future health problems to involve equipping the country, in rural and urban communities, with ambulances, fire brigadiers, and possibly helicopters to help control and prevent health emergencies caused by climate change impacts are an essential factor to contribute to the sampled country’s coping and adaptation strategies towards resilience.

## 4. Discussion

This study principally focused on assessing the preparedness of public health systems in African countries to cope with climate change health hazards. Our results revealed that climate change had been extensively experienced within the sampled African countries, with floods and extreme temperatures being experienced in West Africa, mostly in Ghana and Nigeria. The revelation is confirmed by several studies in the reviewed literature of this study [50,59,64,65]. Drought and extreme temperatures have also been experienced in the Eastern African countries, specifically in Ethiopia and Kenya; meanwhile, the Southern African countries (South Africa and Namibia) also experienced much drought and extreme temperatures. This study shows that extreme temperatures are common impacts of climate change in all the sampled countries. Concurrently, floods and erratic rainfall had also been experienced in all the sampled countries. 

### 4.1. Vulnerability to Climate Change Impacts on Human Health and the Health Systems’ Perception

Extreme weather events experienced in the sampled countries have caused an increase in disease prevalence, such as malaria (endemic in most African countries) and other vector-borne diseases, particularly in Ghana and Nigeria, as the most affected countries. Malnutrition, food insecurity and food-borne diseases are notably occurring in Ethiopia and Nigeria. This confirms the assertion that drought, floods, and extreme temperatures are the most occurring impacts in these countries. Among the sampled countries, Ghana reported the highest effects of climate change on human health. A confirmation of this is seen in a study that applied a series of five hypothetical cases to review the climate impacts on the health and wellbeing of individuals and the population in Sub-Saharan Africa [6]. Additionally, most of the respondents consented to the increase in the trend of climate-induced diseases from the past until now. The result of a study which demonstrated the populations’ impacts from multiple health and social stressors towards extreme weather events is a confirmation to this report [21]. The respondents revealed a significant increase in the trend of diseases and a projection of these diseases in the future if the impacts of climate change are not addressed. Several factors included the government’s inability to collaborate with the relevant stakeholders to ensure adequate training for all health professionals in the health system to address climate change impacts in the sampled African countries, limited funding from the government, insufficient healthcare centres and hospitals, inadequate staff members, inefficient disease surveillance systems, and unavailability of resources, such as medical equipment, rapid response units and their resources (ambulances, fire service van, helicopters in times of flooding and other extreme events). 

Our survey again focused on relationships between climate change impacts and food insecurity. The findings showed that drought, caused by extreme temperatures, has a significant effect on food insecurity and affects the vulnerable population’s health in the sampled countries, most notably in Ethiopia and Nigeria, which respondents perceived to be the two most affected countries. A study conducted in the Horn and Southern Africa, where 14 million lives in Ethiopia, Kenya, Malawi, and Zimbabwe were endangered due to drought, confirmed this report regarding Ethiopia [78]. An ecological study in their findings again indicated that frequent drought increases the population’s food insecurity from 10% to 15% in Ethiopia [54]. From the outcome of this study, the strength of the local capacities in the sampled countries regarding the health system’s eagerness to respond to climate change is weak. According to the respondents’ perceptions, the various intervention programs of the government and N.G.O.s to assist with and address public health issues with food insecurity, vector control and infectious disease prevention in times of climate crises in the sampled countries are inadequate, and those available are ineffective, as seen in the results above. This calls for the sampled African countries to intensify local capacity building in response to their public health vulnerability to climate disasters.

### 4.2. Health System’s Preparedness

Climate change management in the sampled countries needs more attention regarding adaptation and resilience strategies. The justification is found in the diversity of impacts across the continent and each country’s preparedness based on its available resources to deal with the effects. From the studied sampled countries, it was observed that South Africa is making significant progress in its readiness to deal with climate change health impacts. A justification of this is a study that systematically assessed climate change adaptation in the health sector in South Africa and finds significant progress in policy frameworks for climate change, surveillance systems and training curricula for health workers [21].

Several studies argued that each affected nation’s emergency preparedness, response, and recovery activities should widely include human resilience building [79,80,81]. Such action actively needs health professionals with adequate skills and knowledge of handling impacts because healthcare workforces appear at the frontline when it comes to hazards that impact human health. However, the sampled countries’ cases are characterised by low skilled personnel to respond to the impacts. At this stage, the core mission of healthcare structures that provide and secure the public health’s wellbeing is questioning the concerning nations in the most affected areas of the globe.

Education on the climate change impact on human health in the healthcare sector fails to give adequate attention to the roles of the larger eco-systems within which it operates [82]. A study justifies this statement that health professionals’ knowledge of the climate change impact on health should be assessed and suggests the urgent need for education on climate matters [83]. The results of other studies argue that nurses play a vital role in the mitigation, adaptation, and resilience to the climate change impact on human health [84,85,86]. To follow this argument, medical schools in the sampled African countries should find it lawful to integrate climate change into the medical school curriculum to incorporate curricula practice, research, and policy regarding climate change impacts on the environment and human health [87,88,89]. Such action will improve hospital staff’s responsibilities, skills, and competencies regarding responses to climate change’s impact on human health. 

The risk and effects of climate change on mental health are increasing, and intervention to address the issue needs to tackle the problem in a holistic manner [90]. A study reported that child psychological wellbeing is directly and indirectly affected by climate change, and the worst impact occurred in the developing world [91]. These studies confirm our survey, which revealed a lower number of mental health professionals in the health system, addressing the mental health consequences of extreme events on public health. The mental health professional’s role in mitigating climate change impacts and applying strategies to help humans cope with its effects is of more excellent value than reported in a study [91]. 

### 4.3. Available Interventions

This study revealed that the evidence of incorporating climate change into public health intervention is yet to be realised in the sampled countries. However, public health interventions or programs in the sampled countries are well observed. It is a compelling illustration of the local institutions’ weakness in response to climate change impacts on public health. This remark is in line with the result of a study that questioned how reinforcing the public health capacity to respond to extreme events had received less attention [92]. An admitted submission of a study reported the need to incorporate climate change management when implementing public health interventions [93]. South Africa, among the sampled countries, appears to perform with outstanding progress relating to this issue. It challenges other individual African countries in the engagement of long-term interventions in managing climate change impacts on public health. 

### 4.4. Effectiveness of the Available Resources

This study’s critical findings provided clear evidence of ineffective and non-existent disaster relief agencies or programs to assist vulnerable populations and the state’s technical support on developing resources to help public health in case of climate disasters. Most respondents further perceived preventive steps to control future climate change impacts by having effective surveillance systems. When a coordination framework exists between the health systems and the meteorological department to access relevant weather and climate data, the health system plans and implements health programs towards future uncertainties. Again, South African surveillance systems undoubtedly perform better than those of other countries (44.4%).

### 4.5. Policy Application

Policy applications in the sampled African countries regarding improving the present and future generations’ livelihoods in Africa’s climates conditions are lacking. Therefore, we raise the need for the sampled African countries to execute the following:

1. Improve policy frameworks to better consider the many health problems associated with climate change.
2. Improve training provisions to raise awareness among professionals in the health sector about the connections between climate change and health.
3. Improve frameworks for information exchange and the dissemination of best practice across Africa so that successful initiatives dealing with the nexus of climate change and health may be replicated.

Successful application of such policies will help close the gap and address the barriers to successfully implementing adequate coping and adaptation strategies.

#### Advantage and Limitations of the Methodology

The methodology used to perform this study has some advantages. For instance, a survey seemed to be an appropriate way to access a significant group of participants across the African continent as a method. In addition, the technique used permitted the authors to collect data from each participant at a single point in time. Additionally, it provided a picture of the regularity of health hazards in the sampled countries at a precise moment. Moreover, through this methodology, the massive health needs of sampled countries are assessed, providing a sound basis to inform the planning and health authorities about some of the items they may need to consider in allocating health resources. This study’s evidence is also timely since it may help African countries adjust their health systems to cope with climate change. 

However, this study also presented some limitations. Firstly, it only focused on six African countries and did not have the ambition to cover the whole African continent. Additionally, some associations that were identified between climate change and health seemed challenging to interpret since they are not exclusive to climate change. Some structural problems also hinder the handling of various types of diseases. Moreover, because this methodology offers only a one-time measurement of exposure and outcome, it is challenging to evaluate the causal relationship from the data set available. 

## 5. Conclusions

The paper presents an overview of some of the health problems associated with climate change across Africa. A cross-sectional study involving six African countries (Ghana and Nigeria in West Africa, Namibia and South Africa in Southern Africa, Kenya, and Ethiopia in East Africa) analysed the prevailing vulnerability to climate change’s impacts on human health on the one hand and the degree of preparedness of the health systems to cope with them, on the other.

It identifies several trends. First, the levels of knowledge and perceptions about climate variability and public health impacts in the sampled countries are varied. The study also shows that the health impacts of climate change have been extensive, to a greater or at least to some extent, illustrating the fact that they are part of the routine in the investigated countries. Most of the respondents also stated that climate-induced diseases might increase in the future and that future climate changes are not addressed.

The sampled health professionals’ view is that the degree of preparedness of their health systems to deal with the health impacts of climate change in their countries is somewhat limited, a trend that is a reason for concern due to expected increases in the intensity of climate change.

Overall, the responses and examples provided from the sampled countries offer helpful insights into the nature of the nexus of climate change and health, their scope, and possible consequences. It is evident from the study that urgent action is needed to place African countries in a better position to handle the many challenges that climate change poses to them presently and in the future.

The implications of the study are manifold. For instance, it shows that significant differences in the levels of interventions at the country level exist regarding their capacity to handle climate change’s health impacts. There also seems to be a perceived need for disaster relief agencies/organisations to emphasise climate change as part of their work.

Climate change and the effects on human health, the environment, and society will continue to be significant issues in African countries, for which answers need to be found. They pose a challenge to health systems, the governments and health professionals in the coming years. Our findings will be widely shared with health professionals in African countries to support this process to address the barriers to implementing coping and adaptation strategies of climate change impacts on public health. 

There is a perceived need for a greater understanding of climate-related health impacts, which can only be addressed through interdisciplinary research across hierarchical levels and geographical and political boundaries. Because of the challenges that are and will be faced in Africa, it is necessary to sensitise the public and politicians to this topic and support health professionals’ work.

## Figures and Tables

**Table 1:** A systematic review of the sampled countries’ literature to determine their vulnerabilities to the five significant climate change impacts: their exposure to climate drivers, the study location, and the potential resulting outcome from the exposure (see Appendix A). This was developed as a measure of the sampled country’s intensity to climate impacts on human health and the health systems’ preparedness against the impacts through the survey.

Impacts	Author (Year)	Study Period (Year)	City/Country	Study Population	Study Design/Statistical Model	Exposure	Outcome
**Water, sanitation, and water-borne diseases**	[32]	2012	Jamestown/Agbogbloshie (Ghana)	401 Households	Cross-sectional Descriptive, bivariate, and Multivariate	Floods	Reported cases of diarrheal diseases. A strong correlation between flooding and diarrheal disease.
	[30]	1990–2011	North-West (Nigeria)	Morbidity and mortality dataset on cholera cases	Ecological. Stepwise multiple regression, G.A.M.	Min/Max temp, annual Temp and RH	A significant correlation between cholera and annual min/max temperature and rainfall with 1716 deaths from 41,784 cases in 2010 in 18 states.
	[33]	2012–2014	Cape Town (South Africa)	Surveillance database on 58,617 children under five years.	Poisson regression	Min and Max temp	A 32% to 40% increase in diarrhoea incidence at 5 ^o^C increase in Min and Max Temp.
	[34]	2013–2015	Amhara region (Ethiopia)	Retrospective data on children under five years	Ecological Negative Binomial	Average monthly Temp and rainfall	A monthly incidence rate of childhood diarrhoea at 11.4 per 1000 (95% Cl) was significantly associated with mean average temperature and rainfall.
	[72]	1991–1993	Hospital-Based (Malindi). (Kenya).	862 children under five years old	Case-control Binary logistic regression	Rainfall and Temperature	A strong positive correlation between rainfall, temperature, and childhood bloody diarrhoea.
**Vector-borne diseases**.	[38]	1995–2006	National (Ghana)	Reported cases of malaria ranging from 5054 to 347,000 per 100,000	Ecological GLLMM and Local Moran’s *I*	Rainfall, temperature, and humidity	A statistically significant correlation between temperature, humidity, and malaria incidence with a less significant association with rainfall as it only predicted malaria prevalence.
	[39]	1998–2008	Ondo state (Nigeria)	Data on weather variability; cases of malaria in 18 government hospitals	Ecological Poisson multiple regression	Air and sea surface temp.	The occurrence of monthly malaria of 53.4% and 29% at 1 ^o^C increase in air and sea surface temp.
	[40]	1998–2017	Mutale (Limpopo province) (South Africa)	Malaria and climate data	Ecological Spearman correlation SARIMA	Temp. Rainfall RH	A positive significant association malaria incidence and total monthly rainfall, min and max temp., average temp., and mean relative humidity.
	[73]	1989–2009	Amhara, SNNPR, Tigray, Oromia (Ethiopia)	Data on cases of visceral leishmaniasis cases and meteorological data	Ecological Binary and multivariate regression	Annual average Temp. and rainfall	94.7% of *Vl* cases occurred between 20–37 °C of annual average Temp, with fewer cases with increasing rainfall.
	[74]	2004–2014	Baringo county. (Kenya)	Malaria data from 10 health facilities; meteorological data	Ecological Negative binomial regression	Rainfall and Temp.	Rainfall increased malaria transmission across four zones at a time lag of 2 months while temp. increased cases of malaria in riverine and highland zones at time lad of 0 and one month.
**Extreme weather events**	[59]		Secondi-Takoradi (Ghana)	207 heads of households	Mixed cross-sectional Descriptive analysis	Floods	Report of psychological, environmental, and economic problems; disease outbreaks (malaria, cholera, and dysentery).
	(Eludoyin et al., 2013 [65]	1951–2009; 2003–2012	National Akure (Nigeria)	Secondary data Data on the incidence of heat rash.	Ecological Descriptive and correlation analysis	Extreme temperature	Population experience of thermal stress since year 2000 and a significant heat rash among the population between September and December from 2003 to 2012.
	[55]	2011–2012	Ohangwena, Oshana, Omusata (Namibia)	282 households	Cross-sectional	Floods	A remarkable but unspecified number of deaths, injuries, illness from resulting floods.
	[66]	2006–2010	Cape Town, Durban, J’berg (South Africa)	Ambient temperature-all-cause mortality	Case-cross over epidemiological design G.A.M.	Ambient Temp.	Increased temperature above the city-specific threshold significantly increased the general population risk of death (number not specified).
	[75]	2009–2014	National survey (Ethiopia)	55,219 children under five years old	Meta-analysis Bayesian Poisson	Drought	Minimal food-insecure areas showed elevated U5DR compared to stressed food-insecure areas as death rate increases as the prevalence of acute malnutrition increases.
**Nutrition, food security and distribution**	[46]	2016	Bongo District (Ghana)	246 Mother–child pairs (children under 5 years)	Mixed-method cross-sectional Descriptive statistics	Drought	Malnutrition and food insecurity resulted from drought impact, 97.2% being food insecure; children stunting (42.3%), underweight (24.4%) and wasting (17.5%).
	[50]	2015	National data (Nigeria)	Food and crop production index, population density, annual average temp, and rainfall.	Ecological Bivariate correlation and multiple regression	Floods, drought, land use and cover change.	Country’s food deficit due to low agricultural production; hence the country’s dependence on food import. Malnutrition resulting from food insecurity.
	[76]	2013–2014	Dubana and Kwathehle (South Africa)	Children between 24 and 59 months and their caregivers	Cross-sectional Spearman correlation	Summer and winter season	Hunger due to food insecurity was reported in the summer rather than in the winter though their difference in food consumption score was not statistically significant.
	[54]	2014	All regions (Ethiopia)	National F.A.O. dataset	Ecological study	Drought	A frequent drought increased population food insecurity from 10% to 15%.
	[43]	2009–2013	Marsabit district (Kenya)	Children under five years old; 924 households	Panel study Descriptive *z-score*	Drought	Approximately 20% of the children under study were malnourished.
**Mental health and wellbeing**	[60]	2015	Kwaebibrim (History of a flood), West Akyem (no history of a flood) (Ghana)	400 respondents; 200 from each district	Retrospective cohort study Descriptive	Floods	Flood victims more likely to experience symptoms of mental health problems than the non-victims. Reports of significantly higher levels of obsessive compulsion, depression, anxiety, and other global severity indexes.
	[61]	2012	Urban areas affected by floods. (Nigeria)	100 victims of flood-induced crime	Cross-sectional Descriptive analysis	Flooding	Flood-induced crime harms human health and wellbeing with possible effects of anxiety, depression, social dysfunction, and loss of confidence.
	[21]	2018	National (South Africa)	Whole population	Systematic review	Extreme weather events	Population affected by multiple health and social stressors.

Source: From authors.

**Table 2:** The number of respondents from the sampled countries.

Country	Number of Respondents	% of the Total
Ghana	31	25.4%
Nigeria	31	25.4%
South Africa	18	14.8%
Namibia	11	9.0%
Ethiopia	19	15.6%
Kenya	12	9.8%
Total	122	100%

Source: From authors.

**Table 3:** Respondents’ workplaces.

Country	Government Agencies	Higher Institutions	Non-Governmental Agencies	Research Institutions	Total within the Country
Ethiopia	21.0%	47.4%	31.6%	0.0%	100.0%
Ghana	46.6%	26.7%	6.7%	20.0%	100.0%
Kenya	27.2%	36.4%	0.0%	36.4%	100.0%
Namibia	45.4%	27.3%	27.3%	0.0%	100.0%
Nigeria	21.2%	45.5%	21.2%	12.1%	100.0%
South Africa	22.2%	44.4%	5.6%	27.8%	100.0%
% of the total	30.3%	38.5%	15.6%	15.6%	100.0%

Source: From authors.

**Table 4:** Respondents’ understanding of climate change and human health impacts.

Country	Yes, a Lot	Yes, Some	Yes, Little	Total within the Country
Ghana	19.4%	61.3%	19.3%	100%
Nigeria	42.0%	54.8%	3.2%	100%
South Africa	22.2%	44.5%	33.3%	100%
Namibia	0.0	72.7%	27.3%	100%
Ethiopia	52.6%	42.1%	5.3%	100%
Kenya	16.7%	75.0%	8.3%	100%
% of Total	28.7%	56.6%	14.7%	100%

Source: From authors.

**Table 5:** Sampled countries’ diseases trend from the past until now as perceived by respondents.

Country	Increased	No Changes	Decreased	I Do Not Know	Total within the Country
Ghana	71.0%	16.1%	9.7%	3.2%	100%
Nigeria	80.6%	19.4%	0.0%	0.0%	100%
South Africa	66.7%	16.7%	5.5%	11.1%	100%
Namibia	81.8%	9.1%	9.1%	0.0%	100%
Ethiopia	68.5%	10.5%	10.5%	10.5%	100%
Kenya	75.0%	0%	16.7%	8.3%	100%
% of Total	73.8%	13.9%	7.4%	4.9%	100%

Source: From authors.

**Table 6:** Respondents’ perceptions of the health system’s preparedness in dealing with the health impacts of climate (CI) change in sampled countries. Numbers are assigned to their respective survey questions on top of the table. Even though percentages summed up to 100, this table’s options are of greater importance to this study (see Appendix A). This applies to all other subsequent tables with similar instances. All percentages are recorded within each country’s number of respondents to identify specific features better.

Country	1. Preparedness a Priority in the Country 2. Experts to Deal with Climate Impacts (CI) 3. Healthcare Delivery Systems to Address CI. 4. Addressing Mental Illness Caused by Extreme Events. 5. Enough Training of Health Professionals 6. Enough Healthcare Personnel	Yes, but a Little	No, Not at All
	7. Preparedness to Respond to Extreme Events	Fairly Well Prepared	Not so Much Prepared
**Ghana**	1.	58.1%	22.6%
	2.	61.3%	22.6%
	3.	77.4%	6.5%
	4.	83.9%	12.9%
	5.	67.7%	16.1%
	6.	83.9%	12.9%
	7.	45.2%	48.4%
**Nigeria**	1.	58.1%	25.8%
	2.	51.6%	22.6%
	3.	64.5%	29.0%
	4.	67.7%	19.4%
	5.	67.7%	22.6%
	6.	61.3%	25.8%
	7.	25.8%	71.0%
**South Africa**	1.	33.3%	16.7%
	2.	66.7%	5.6%
	3.	55.6%	27.8%
	4.	50.0%	27.8%
	5.	72.2%	16.7%
	6.	55.6%	16.7%
	7.	44.4%	44.4%
**Namibia**	1.	45.5%	27.3%
	2.	45.5%	27.3%
	3.	54.5%	9.1%
	4.	63.6%	27.3%
	5.	36.4%	45.5%
	6.	45.5%	45.5%
	7.	19.2%	72.7%
**Ethiopia**	1.	57.9%	10.5%
	2.	78.9%	0.0%
	3.	94.7%	0.0%
	4.	73.7%	21.1%
	5.	52.6%	42.1%
	6.	52.6%	36.8%
	7.	47.4%	47.4%
**Kenya**	1.	41.7%	41.7%
	2.	66.7%	16.7%
	3.	75.0%	8.3%
	4.	66.7%	25.0%
	5.	50.0%	33.3%
	6.	50.0%	25.0%
	7.	50.0%	47.1%

Source: From authors.

**Table 7:** Sampled countries’ intervention programs to deal with the health impacts of climate change as perceived by respondents.

Country	Control of Climate Impacts 1. Water-Borne Diseases Control 2. Vector-Borne Diseases and Vector Control 3. Nutrition, Food Security and Distribution Organisations 4. Extreme Events Adaptation	Available, but Not Effective	Not Available
**Ghana**	1. 2. 3. 4.	77.4% 54.8% 74.2% 58.1%	3.2% 9.7% 9.7% 22.6%
**Nigeria**	1. 2. 3. 4.	45.2% 48.4% 64.5% 54.8%	16.1% 16.1% 12.9% 22.6%
**South Africa**	1. 2. 3. 4.	66.7% 27.8% 55.6% 38.9	0.0% 27.8% 0.0% 27.8%
**Namibia**	1. 2. 3. 4.	63.6% 18.2% 54.5% 81.8%	0.00% 9.1% 9.1% 0.0%
**Ethiopia**	1. 2. 3. 4.	52.6% 36.8% 36.8% 52.6%	00% 10.5% 10.5% 5.3%
**Kenya**	1. 2. 3. 4.	58.3% 50.0% 50.0% 50.0%	16.7% 8.3% 25.0% 25.0%

Source: From authors.

**Table 8:** Respondents’ perception of sampled country’s disaster relief agencies/organisations helps victims of extreme food distribution and shelter victims.

Country	Percentages of ‘Not Enough’ as Reported by Respondents
Ghana	77.4%
Nigeria	64.5%
South Africa	50.0%
Namibia	81.8%
Ethiopia	78.9%
Kenya	58.3%

Source: From authors.

**Table 9:** Technical support from sampled countries’ government and the needed funding/budget in developing preparedness plans and communicating with the public about climate change’s health effects, as perceived by respondents.

Country	1. Climate Change Incorporated into Public Health Interventions	Yes, but a Little	No, Not at All
	2. Technical Support from the Government		
	3. Budget/Funding Needed	Perceived Percentage Funding/Budget Needed	
**Ghana**		83.9%	12.9%
	**1.**	77.4%	16.1%
	**2.**	90.3%	
**Nigeria**	**1.**	41.9%	32.3%
	**2.**	64.5%	22.6%
	**3.**	**96.8%**	
**South Africa**	**1.**	38.9%	22.2%
	**2.**	22.2%	44.4%
	**3.**	**77.8%**	
**Namibia**	**1.**	54.5%	27.3%
	**2.**	27.3%	27.3%
	**3.**	**90.9%**	
**Ethiopia**	**1.**	63.2%	21.1%
	**2.**	47.4%	42.1%
	**3.**	**89.5%**	
**Kenya**	**1.**	58.3%	25.0%
	**2.**	50.0%	25.0%
	**3.**	**91.7%**	

Source: From authors.

**Table 10:** Respondents’ perceptions about the health system’s available resources and those needed to curb climate change (CC) impact human health in the sampled countries.

Country	1. Available Resources to Deal with Health Impact of CC	Limited Availability (%)
	2. Expand and Construct More Hospital and Health Centres. 3. Additional Staffs Needed. 4. Staff Training 5. Medical Equipment Needed. 6. Good Surveillance System 7. Emergency Response Unit/Resources Needed	Needed Resources Perceived (% within a Country)
**Ghana**	1.	61.9%
	2.	83.9%
	3.	74.2%
	4.	83.9%
	5.	83.9%
	6.	77.4%
	7.	90.3%
**Nigeria**	1.	61.3%
	2.	80.6%
	3.	77.4%
	4.	90.3%
	5.	90.3%
	6.	87.1%
	7.	93.5%
**South Africa**	1.	66.7%
	2.	61.1%
	3.	55.6%
	4.	94.4%
	5.	55.5%
	6.	44.4%
	7.	66.7%
**Namibia**	1.	90.9%
	2.	66.6%
	3.	72.7%
	4.	81.8%
	5.	81.8%
	6.	72.7%
	7.	63.6%
**Ethiopia**	1.	68.4%
	2.	78.9%
	3.	89.5%
	4.	94.7%
	5.	84.2%
	6.	78.9%
	7.	84.4%
**Kenya**	1.	75.0%
	2.	75.0%
	3.	66.7%
	4.	91.7%
	5.	75.0%
	6.	75.0%
	7.	66.7%

Source: From authors.

## Data Availability

All original data are available for consultation.

## Appendix A

The questionnaire aims to gather the information that can help assess the preparedness of the health systems in African countries to cope with the health hazards posed by climate change. Six African countries are under study: Ghana, Nigeria, Namibia, South Africa, Ethiopia, and Kenya. The survey seeks to assess the knowledge and perceptions of health professionals from these African countries on the anomalies of climate change on human health, to what extent the country is vulnerable to climate change impacts and how sustainable the preparedness is to deal with unforeseen climate impacts and identify best practices in dealing with climate change impacts on human health.

**Background Information**

Gender: Male…………… Female……… Prefer not to say………..

Age: ………………..

Country: Ghana…….. Nigeria……. South Africa……… Namibia……. Ethiopia…. Kenya………

City/region: ……….

Respondent’s workplace

N.G.O.…………………………………….

Government Agency………………….

Higher educational institution………..

Other……………………………………

Position of respondents

Answer…………………

**Knowledge and perception of climate change and health impacts**

1. Where do you obtain your climate/weather data?

Meteorological agent…………………

Radio/television……………………….

Internet…………………………………

Other……………………………………

2. Do you know the potential public health impacts of climate change?

Yes, a lot…………………..

Yes, some…………………

Yes, but just a little……….

3. If yes, would you please list down the potential health impacts of climate change that you know?

Your answer…………………………………………………………………………………

**Public health’s vulnerability to climate change impacts in the country**

4. Has the country experienced climate change in the past years?

Yes, very much so………………….

Yes, a little…………………………..

No, not at all…………………………

5. If yes, which of the following have been observed as impacts? Can you please tick and rank only the observed impacts according to their magnitude of occurrence?

**Climate Impacts**	**Most Frequent**	**More Frequent**	**Frequent**	**Less Frequent**	**Least Frequent**
Floods					
Drought					
Wildfires					
Extreme temperature					
Sea level rise					
Change in Precipitation					
Increase carbon dioxide					
Other					

6. Have you observed any health problems associated with these impacts in the country?

Yes, very much so…………………..

Yes, a little…………………………..

No, not at all………………………..

7. If yes, which of the following public health problems have been observed in your country/regions? Can you please tick and rank only the observed effects according to their prevalence?

	**Most Prevalent**	**More prevalent**	**Prevalent**	**Less Prevalent**	**Least Prevalent**
Malaria and other vector-borne diseases					
Diarrhoea, water-borne diseases, and sanitation					
Malnutrition, food-borne and food insecurity					
Climate-induced mental health consequences					
Extreme weather events					

8. What do you think about the trend of the diseases/ situations above, from the past until now?

Increased…………………………………….

Decreased……………………………………

No change, prevailing……………………..

I do not know………………………………

9. In the next 15–20 years, what do you think will likely trend the country’s health mentioned above problems following future climate change?

Increase………………………………………

Decrease……………………………………..

Stabilise………………………………………

**Preparedness**

10. Is preparedness to deal with climate change’s public health impacts a priority in the country/region?

Yes, very much so…………………..

Yes, a little……………………………

No, not at all………………………….

11. Are there enough experts/professionals in the country/region who can assess potential public health impacts associated with climate change?

Yes, enough……………………………..

Yes, but not enough…………………….

No, not at all……………………………..

12. Are there enough health care delivery systems in your country/regions —including the hospitals, health centres, and clinics, to address climate change impact on health?

Yes, enough…………………………………

Yes, but not enough……………………….

No, not at all…………………………………

13. Are there enough mental health professionals/centres in the country/region to address extreme public health’s cognitive consequences?

Yes, enough…………………………………

Yes, but not enough………………………..

No, not at all………………………………..

14. Do public health professionals in the country/region have enough training on health risks interventions related to climate change?

Yes, enough…………………………….

Yes, but not enough……………………

No, not at all…………………………….

15. Do hospitals, health centres and clinics in the country/region have enough health personnel to deal with health problems associated with climate change?

Yes, enough…………………………….

Yes, but not enough……………………

No, not at all……………………………

16. How prepared are the health systems in your country to respond to extreme events (floods, drought, wildfires, extreme temperature?

Very well prepared………………………..

Fairly well prepared………………………

Not so much prepared………………….

Not at all…………………………………..

**Interventions**

17. Do you think climate change is incorporated into public health intervention?

Yes, very much so……………………..

Yes, a little………………………………

No, not at all…………………………….

18. Do you have technical support from the government forecasting local effects, developing preparedness plans, and communicating with the public about climate change’s health effects?

Yes, very much so………………………...

Yes, a little…………………………………..

No, not at all………………………………..

19. Do you know of the following public health interventions/programs in your country/region to deal with climate change impacts; how effective or ineffective these interventions/programs are if they exist?

**Intervention programs**	**Yes, and Effective**	**Yes, but not Effective**	**No, they do not Exist**
Water availability, sanitation, and water-borne diseases control			
Vector control			
Nutrition, food safety and distribution during a famine			
Extreme events adaptation			

20. If yes, what are some of the interventions/programs to deal with climate change impacts in your country/region? Please give your answers according to the numbers provided on a separate line. Do not repeat the options, please (e.g., 1…..., 2....)

1. water availability, sanitation and water-borne; 2. Vector control

21. Are there enough disaster relief agencies/programs to assist public health with food distribution in case of climate disasters?

Yes, enough…………………………

Yes, but not enough………………..

No, Not at all………………………..

22. If yes, can you please give examples of those existing programs/agencies in question 21.?

Your answer…………………………….

23. Do you think the country’s public health systems are building resilience to unforeseen climate change impacts?

Yes, I strongly think so………………………

Yes, but not enough……………………….

No, I do not think so…………………………

**Resources Availability**

24. Do the health systems have available resources to deal with climate change impacts on human health?

Yes, Largely available…………………

Yes, to some extent……………………

Yes, but limited…………………………

No, not at all…………………………….

25. Which other resources below do you think are needed by the health systems to help curb climate change’s health impacts?

Expanding and construction of more health centres

Additional staff

Staff training

medical equipment

Budgets/Money/funding

Surveillance systems

Quick response or emergency relief resources (ambulances, helicopters, firefighting van etc.)

Other:
